# Phosphate Deprivation Can Impair Mechano-Stimulated Cytosolic Free Calcium Elevation in *Arabidopsis* Roots

**DOI:** 10.3390/plants9091205

**Published:** 2020-09-15

**Authors:** Elsa Matthus, Nicholas H. Doddrell, Gaëtan Guillaume, Amirah B. Mohammad-Sidik, Katie A. Wilkins, Stéphanie M. Swarbreck, Julia M. Davies

**Affiliations:** 1 Department of Plant Sciences, University of Cambridge, Downing Street, Cambridge CB2 3EA, UK; ematthus@hotmail.com (E.M.); nicholas.doddrell@emr.ac.uk (N.H.D.); gaetanguillaume@laposte.net (G.G.); amirahbmsa@gmail.com (A.B.M.-S.); kaw67@cam.ac.uk (K.A.W.); stephanie.swarbreck@niab.com (S.M.S.); 2 NIAB EMR, New Road, East Malling ME19 6BJ, UK; 3 The John Bingham Laboratory, NIAB, 93 Lawrence Weaver Road, Cambridge CB3 0LE, UK

**Keywords:** *Arabidopsis*, calcium, FERONIA, mechano, phosphate, root, skew, touch

## Abstract

The root tip responds to mechanical stimulation with a transient increase in cytosolic free calcium as a possible second messenger. Although the root tip will grow through a heterogeneous soil nutrient supply, little is known of the consequence of nutrient deprivation for such signalling. Here, the effect of inorganic phosphate deprivation on the root’s mechano-stimulated cytosolic free calcium increase is investigated. *Arabidopsis* *thaliana* (cytosolically expressing aequorin as a bioluminescent free calcium reporter) is grown in zero or full phosphate conditions, then roots or root tips are mechanically stimulated. Plants also are grown vertically on a solid medium so their root skewing angle (deviation from vertical) can be determined as an output of mechanical stimulation. Phosphate starvation results in significantly impaired cytosolic free calcium elevation in both root tips and whole excised roots. Phosphate-starved roots sustain a significantly lower root skewing angle than phosphate-replete roots. These results suggest that phosphate starvation causes a dampening of the root mechano-signalling system that could have consequences for growth in hardened, compacted soils.

## 1. Introduction

Mechanical stimulation can alter root growth and development [1,2,3,4,5]. Considering the cellular level, it causes a transient increase in cytosolic free calcium ([Ca^2+^]_cyt_) (the “touch response”) as a second messenger that governs transcriptional responses [3,6,7]. Plasma membrane (PM) mechano-sensitive channels or components of channel complexes may be involved in the [Ca^2+^]_cyt_ response, mediating Ca^2+^ influx. Candidates include MCA1 (Mid1-Complementing Activity1, [1]), MCA2 [8], OSCA1 (reduced hyperosmolality-induced [CA^2+^]_cyt_ increase1, [9,10]), MSL10 (MscS-like10, [11]) and DEK1 (Defective Kernel 1, [12,13]). Alternatively, PM Ca^2+^ channels governed by PM receptor-like kinases (that may or may not be wall-associated) would be competent to elevate [Ca^2+^]_cyt_ [3,14,15]. Regarding roots, mechanical stimulation can evoke a spatially complex [Ca^2+^]_cyt_ elevation and sensitivity varies along the root [2,3,16,17]. This [Ca^2+^]_cyt_ signal lies downstream of the PM kinase FERONIA (FER), can be decoded by Calmodulin-like (CML) proteins (CML12 and CML24), and has been linked to mechanically-induced changes in root system architecture [2,3,18]. Thus far, the complete signal transduction cascade from change in [Ca^2+^]_cyt_ to response at the whole root level is far from being resolved.

As the root tip grows through the soil, it will experience both mechanical stimulation and a heterogeneous nutrient supply. Few studies have examined whether nutrient supply can influence [Ca^2+^]_cyt_ signalling but, recently, it was shown that inorganic phosphate (Pi) deprivation can alter the patterns of abiotic stress-induced [Ca^2+^]_cyt_ elevation of *Arabidopsis thaliana* roots [19,20]. These can be reversed by Pi re-supply [19]. It was not clear from these studies whether Pi deprivation could alter the root’s mechanically stimulated [Ca^2+^]_cyt_ elevation. This could potentially be important as agricultural soils are commonly both compacted and Pi-deficient and use of Pi-fertiliser is not sustainable [4]. Here, the effect of Pi deprivation on the [Ca^2+^]_cyt_ touch response of *Arabidopsis thaliana* roots is examined using cytosolic (apo)aequorin as a luminescent [Ca^2+^]_cyt_ reporter. Additionally, the ability of roots to skew as an output of mechanical stimulation and [Ca^2+^]_cyt_ signalling is tested [3,18]. Skewing is the deviation from vertical that occurs when a root is grown on a vertical or inclined surface [3,21]. That skewing persists in microgravity points to mechanical stimulation experienced by the root tip as a key component of this growth phenomenon [22]. *Arabidopsis* mutants for FER and CML24 have an enhanced skewing angle [3,18]. As the fer mutant has an impaired [Ca^2+^]_cyt_ touch response, the [Ca^2+^]_cyt_ signal might negatively regulate skewing. Here, Pi deprivation impairs the root’s [Ca^2+^]_cyt_ touch response but also results in a *lower* skewing angle, in contrast to fer [3]. These results suggest that Pi starvation causes a dampening of the root mechano-signalling system and that a full mechanically stimulated [Ca^2+^]_cyt_ response acts as a positive regulator of skewing.

## 2. Results

### 2.1. Phosphate Starvation Contributes to Impaired [Ca^2+^]_cyt_ Elevation in Response to Mechanical Stimulation

**Figure 1:**
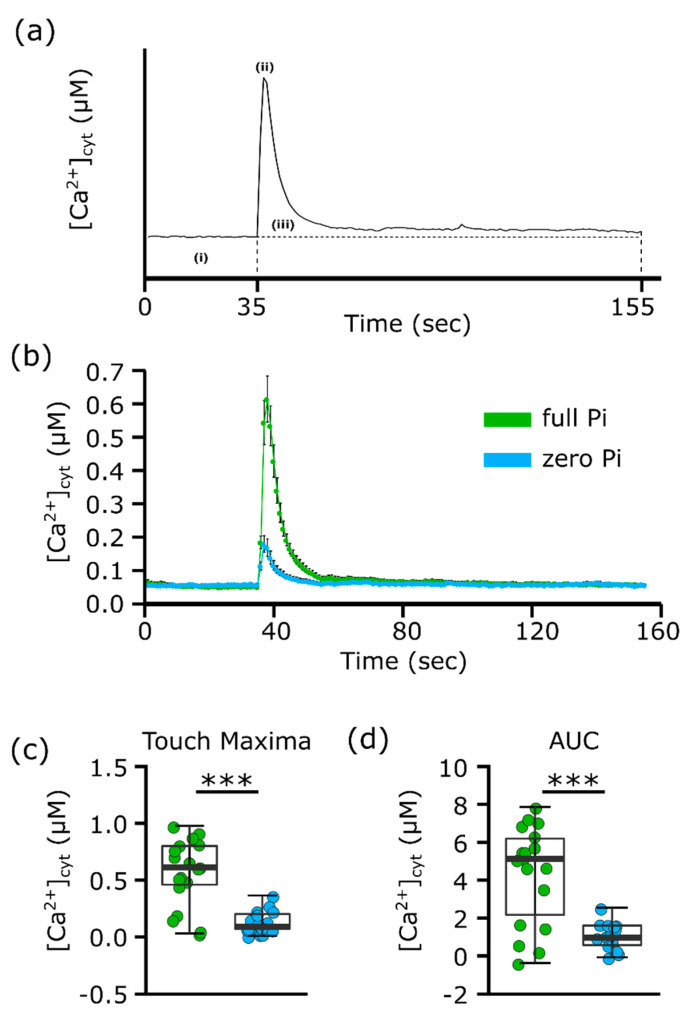
Mechano-stimulated root [Ca^2+^]_cyt_ increase is impaired in Pi-starved root tips. (**a**) Schematic of [Ca^2+^]_cyt_ analysis of aequorin time-course data. Each value was calculated with the average baseline value (i) subtracted. Touch peak was the highest value of the touch response due to mechanical stimulus from the solution application (ii; 35–155 s). Total [Ca^2+^]_cyt_ accumulation (iii) was estimated by integrating the area under the curve (AUC). (**b**) Individual root tips (1 cm) of 11-day old *Arabidopsis* seedlings in ± Pi liquid growth medium were mechanically stimulated by application of full Pi or zero Pi liquid growth medium. There was no significant difference in pre-test baseline [Ca^2+^]_cyt_. Time course trace represents mean ± standard error of mean (SEM) from three independent trials, with 17–18 individual root tips averaged per data point. (**c**) Time course data were analysed for touch maxima relative to the baseline, with each dot representing an individual data point. The thick middle line denotes the median, separating the upper and lower half of the data; the hinges (box outline) denote median of the upper and the lower half of the data, respectively; the bars denote entirety of data excluding outliers. (**d**) Time course data were analysed for AUC beyond the baseline concentration. Significance levels: *** *p* < 0.001, Welch two sample *t*-test.

The [Ca^2+^]_cyt_ elevation caused by mechanical stimulation of Pi-replete and Pi-starved Col-0 individual root tips expressing cytosolic (apo)aequorin was first determined (Figure 1a). Excised root tips (1 cm) were challenged with full- or zero-Pi liquid medium, corresponding to the solid growth medium used (Figure 1b). The mechanical stimulation caused by such liquid addition caused a “touch response” [3]; a rapid monophasic and transient increase in [Ca^2+^]_cyt_ with a discernible “peak” (three independent trials, with 17–18 individual root tips per growth condition; Figure 1a,b). The peak [Ca^2+^]_cyt_ touch response of full Pi-grown root tips was significantly greater than that of Pi-starved root tips (full Pi-grown 0.57 ± 0.07 µM; zero Pi-grown 0.13 ± 0.02 µM, *p* < 0.001; Figure 1b,c). Full Pi-grown root tips also had a significantly higher area under the curve (AUC; Figure 1a), which is an estimate of total [Ca^2+^]_cyt_ mobilised (*p* < 0.001, Figure 1d).

**Figure 2:**
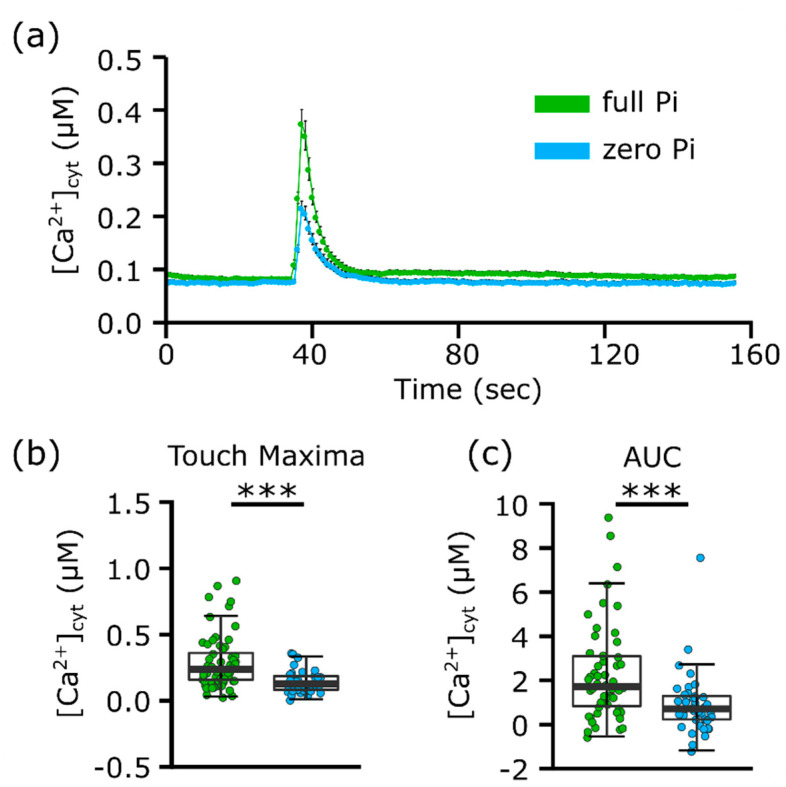
Excised Pi-starved whole roots have an impaired [Ca^2+^]_cyt_ touch peak. (**a**) Individual excised roots of 11-day old seedlings in ± Pi liquid growth medium were mechanically stimulated by application of full Pi or zero Pi liquid growth medium. Time course trace represents mean ± SEM from five independent trials, with 41–61 individual roots averaged per data point. (**b**) Time course data were analysed for touch maxima, with each dot representing an individual data point. Boxplot middle line denotes median. (**c**) Time course data were analysed for AUC. Significance levels: *** *p* < 0.001, Welch two sample *t*-test.

Wounding can influence gene expression even at the level of a neighbouring cell [23]. Even though root tips had an overnight recovery from excision, the wounding site was relatively close to the likely site of mechano-perception at the apex. To reduce the possible influence of wounding on the response to mechanical stimulation, roots were excised at the junction with the stem. The distance from the wound site to the apex was approximately 5 cm for full Pi-grown root and 3 cm for Pi-starved. The impairment in the [Ca^2+^]_cyt_ touch response of zero Pi-grown roots remained evident in 5 independent trials (Figure 2a). The peak [Ca^2+^]_cyt_ touch response of full Pi-grown roots was significantly greater than that of zero Pi-grown roots (full Pi-grown 0.3 ± 0.03 µM; zero Pi-grown 0.16 ± 0.01 µM, *p* < 0.001; Figure 2b), as was the AUC (*p* < 0.001; Figure 2c). Thus, the impairment in the [Ca^2+^]_cyt_ touch response in excised root tips of Pi-starved plants was recapitulated in whole Pi-starved roots and appears less likely to be a consequence of the excision site.

### 2.2. Phosphate Starvation Results in Impaired Root Skewing

**Figure 3:**
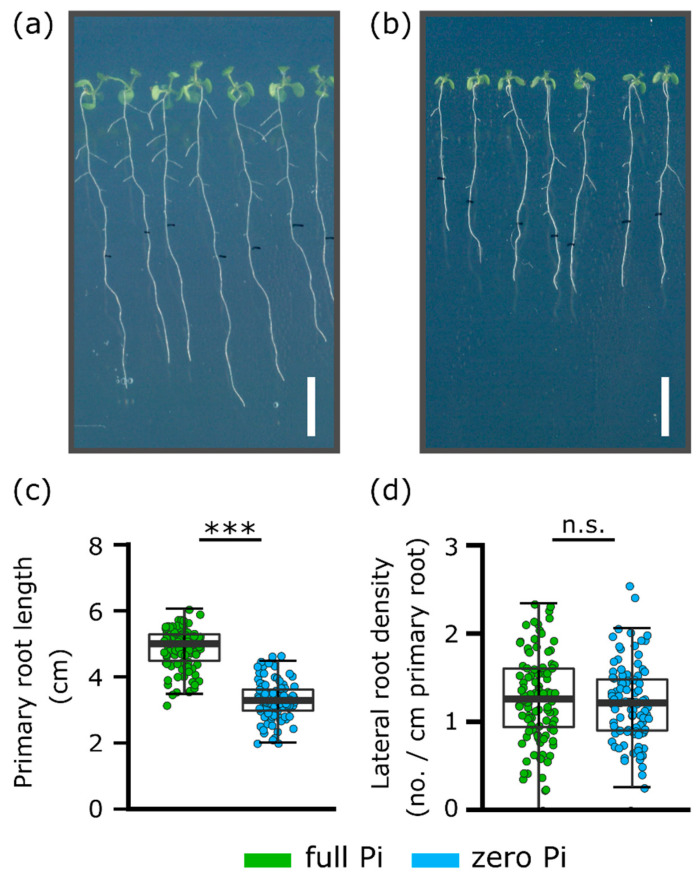
Pi-starved roots suffered growth inhibition. *Arabidopsis* Col-0 roots were grown vertically for 11 days on (**a**) full or (**b**) zero Pi medium. (**c**) Primary root length. (**d**) Lateral root density. Mean ± SEM from three independent trials with 100–106 plants. Significance levels: n.s., not significant; *** *p* < 0.001, Welch two sample *t*-test. Scale bar in (**a**) and (**b**) = 1 cm. Scans were taken viewed from the back of the plate.

The impaired [Ca^2+^]_cyt_ touch response of Pi-starved roots implies the downstream consequences of mechano-signalling also might be affected. Growth on a vertical surface causes roots to skew (deviate from the vertical) and it is held that mechanical stimulation of the root apex is a contributory factor, with [Ca^2+^]_cyt_ signalling implicated as a negative regulator [3,18,21,24]. It, therefore, was hypothesised that Pi-starved roots also may have an enhanced skewing phenotype, in common with the fer mutant [3]. Roots grown for 11 days on zero Pi medium were significantly shorter than those grown on full Pi (full Pi grown roots: 5.0 ± 0.06 cm, zero Pi grown roots: 3.3 ± 0.06 cm, Figure 3a–c, *p* < 0.001), consistent with effective Pi-starvation growth arrest [25] although, at this stage, there was no effect on lateral root density (Figure 3d; three independent experiments with 100–106 plants per growth condition).

**Figure 4:**
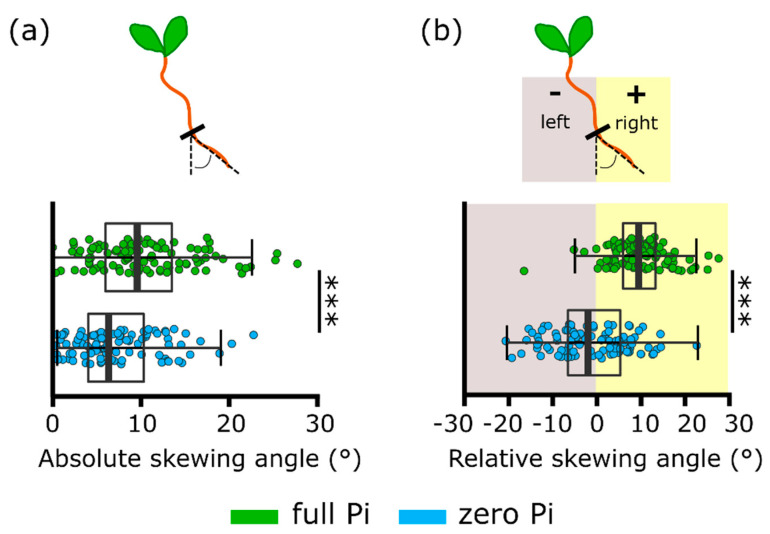
Pi-starved plants have a reduced rightward skewing angle. (**a**) Absolute skewing angle (value of the skew relative to vertical, regardless of direction) of roots after 11 days of vertical growth on full or zero Pi medium. (**b**) Relative skewing angle (+ to the right, − to the left, when viewed from the back of the plate). Mean ± SEM from three independent trials shown in Figure 3, with 100–106 plants per growth condition. Significance levels: *** *p* < 0.001, Welch two sample *t*-test.

Estimation of absolute root skewing angle (value of the skew relative to vertical, regardless of direction) revealed that Pi-starved roots had a significantly lower skew (full Pi-grown 10.4 ± 0.6°; zero Pi-grown 7.3 ± 0.5°, Figure 4a; *p* < 0.001). When the direction of the skew was considered (relative angle), roots grown on full Pi medium had a rightward skew but those of Pi-starved roots skewed significantly less to the right (full Pi-grown 10.0 ± 0.6°; zero Pi-grown −1.3 ± 0.9°, Figure 4b; *p* < 0.001).

**Figure 5:**
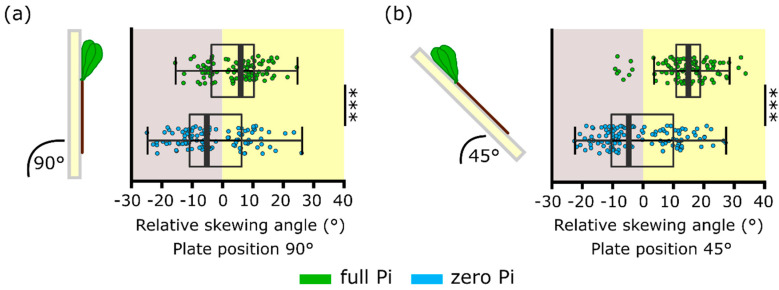
Pi-starved roots do not increase their skewing angle in response to inclined growth. (**a**) Roots were grown vertically on full or zero Pi medium. (**b**) Roots were grown at a 45° incline on full or zero Pi medium. Mean ± SEM from three independent trials with 106–121 plants. Significance levels: *** *p* < 0.001, Welch two sample *t*-test.

During a separate set of experiments, plants also were grown at a 45° incline. This is a condition held to increase the mechanical stimulation at the root tip and results in a greater skewing angle [21,24,26]. Here, roots grown either vertically (90°) or on a 45° incline on full Pi medium still displayed a significantly greater rightward skew than those grown on zero Pi at either angle (Figure 5a,b; *p* < 0.001). When grown at 45°, full Pi-grown roots skewed significantly more rightward than those grown vertically (full Pi roots grown at 45° plate position: 14.3 ± 0.8°, full Pi roots grown at 90° plate position: 4.5 ± 0.8°, *p* < 0.001). This indicates that the incline was effective in promoting skewing. To contrast, there was no significant difference between zero Pi-grown roots grown vertically or on a 45° incline (zero Pi roots grown at 45° plate position: −1.5 ± 1.1°, zero Pi roots grown at 90° plate position: −2.8 ± 1.1°, *p* = 0.43). This indicates an impairment in Pi-starved roots to sense and/or respond to the increased mechanical stimulation resulting from the inclined growth medium.

## 3. Discussion

Roots growing in the soil encounter a heterogeneous environment where they are exposed to both mechanical stimulation and nutrient deficiencies. Here we show that Pi deficiency can affect the root’s response to mechanical stimulation both at the cellular level (through altered [Ca^2+^]_cyt_ elevation) and at the whole root level (root skewing). The mechanistic basis of a dampened [Ca^2+^]_cyt_ touch response of Pi-starved roots could have a variety of origins. These might include the effects of wall stiffening that occurs during deprivation, or PM lipid remodelling [25,27,28,29,30,31] acting to lessen the stimulus. Modulation of wall structure could affect the activity of PM kinases such as FER. A recent study on Pi-starved rice shoots found an increased level of FER transcript but this could be countered by increased internalisation of the protein [31]. Abundance of this pivotal kinase, therefore, could underpin the root’s touch [Ca^2+^]_cyt_ response during Pi starvation. Downregulation of mechano-sensitive PM Ca^2+^ influx pathways (e.g., MCA1 or OSCA1) also might occur. However, in previous studies on Pi starvation of *Arabidopsis* roots, no putative Ca^2+^ channel subunits were found to be downregulated at the protein level [30,32,33]. The only putative Ca^2+^ channel subunit found to be significantly transcriptionally downregulated after Pi starvation was CNGC15 (Cyclic Nucleotide-Gated Channel15), but this was transient [34]. CNGC15 co-localises in the nucleus with the cation channel DMI1 (Does not Make Infections1) and could be involved in nuclear Ca^2+^ signalling for auxin homeostasis in *Arabidopsis* roots [35]. It also should be considered that any dampened [Ca^2+^]_cyt_ response could be the effect of an upregulated PM Ca^2+^ efflux transporter. Early work with tomatoes reported a strong upregulation of LCA1 (*Lycopersicon* Calcium ATPase1) encoding a root Ca^2+^-ATPase, under Pi starvation [36]. Regarding *Arabidopsis*, upregulation of Cation Exchanger3 (CAX3), CAX7 and Autoinhibited Ca^2+^-ATPases13 (ACA13) was detected at the transcriptional level [34,37,38]. However, transcriptional upregulation did not translate into higher protein abundance [30,32]. Post-translational modification of Ca^2+^ transporters also might occur under Pi deprivation.

An impaired mechanically stimulated [Ca^2+^]_cyt_ response could manifest in an altered growth response to mechanical stimulation. Here, root skewing was used as a preliminary test of “phenotypic output” from mechanical stimulus and [Ca^2+^]_cyt_ elevation [3,18]. Pi deprivation was effective in arresting primary root growth [25] but did not cause the lateral root proliferation found by Williamson et al. [39]. Not all studies have reported increased lateral root density under Pi deprivation [40] and deprivation can lead to time-dependent suppression of laterals [41]. It was expected that zero Pi-grown roots would have an enhanced skewing response because the fer mutant has an impaired mechanically stimulated [Ca^2+^]_cyt_ response, but an enhanced skewing phenotype under normal growth conditions, suggesting that the [Ca^2+^]_cyt_ signal negatively regulates skewing [3]. While no case can be made for causality, it is intriguing that zero Pi-grown roots skewed less than Pi-grown roots and failed to respond to the additional mechanical stimulation afforded by growth on an inclined plate. This would be consistent with a need for a full mechanically stimulated [Ca^2+^]_cyt_ response as a positive regulator of skewing, rather than a negative regulator. Downstream components of the [Ca^2+^]_cyt_ pathway are likely to include CMLs and IQD (IQ67 Domain) proteins, with microtubules as ultimate targets thus affecting cellulose synthases to effect root growth [18,42]. However, regulation of root skewing is complex, involving elements of stress and hormonal signalling plus cell wall modifications [21,24,26,43], making cause and effect difficult to establish. Indeed, the impaired touch [Ca^2+^]_cyt_ response of fer was determined for epidermal cells [3] and may not relate to touch-induced skewing that can be envisaged to emanate from apical responses. More genes contributing to *Arabidopsis* skewing are being identified and, of these, ASN1 (Asparagine Synthetase [Glutamine-hydrolysing1], involved in sensing sucrose starvation and darkness) is downregulated when roots are grown at 45° but upregulated under Pi deprivation [26,34]. Simplistically, if ASN1 were a negative regulator of skewing, then this expression pattern could link to the reduced skewing responses found here for zero Pi-grown roots.

The [Ca^2+^]_cyt_ touch response measured with aequorin can be highly variable [44]. Two previous studies on *Arabidopsis* Pi-starved root tips reported either a significant diminution of the [Ca^2+^]_cyt_ touch response or no effect [19,20]. Nitrogen deprivation had no effect, suggesting a nutrient-specific phenomenon [19]. Here, both root tips and whole roots of zero Pi-grown *Arabidopsis* had a significantly lower [Ca^2+^]_cyt_ touch response than Pi-replete samples in terms of peak elevation and total [Ca^2+^]_cyt_ mobilised (Figure 1 and Figure 2). These further data sets suggest that Pi deprivation may well alter perception and/or signalling of mechanical stimulation and warrant further investigation, such as how quickly Pi resupply could reset the system [19]. No study as yet has addressed the impact of Pi starvation on the mechanically stimulated [Ca^2+^]_cyt_ response of stem or leaf tissues, which may be relevant to wind stress. Future studies would need to deploy [Ca^2+^]_cyt_ reporters capable of greater spatial resolution, such as Fura-2, the Yellow Cameleons, or GCaMP3. Ideally, roots would be growing vertically and not excised. Additionally, the mechanical stimulus needs to be directed at specific parts of the root with defined force relevant to those experienced in soil [17]. Further studies on responses of Pi-deprived roots to mechanical stimulation, such as obstacle contact or growth into hard agar [1] or soils [45], are now needed. It also would be interesting to determine whether Pi-starved roots have aberrant touch-induced hormonal responses, such as impaired regulation of gibberellins, ethylene and jasmonic acid, and whether these relate to touch-induced changes in root system architecture [5,46,47]. Such studies could be relevant to the challenge of breeding crops for compacted and Pi-poor soil [4,45].

## 4. Materials and Methods

### 4.1. Plant Material and Growth Conditions

*Arabidopsis thaliana* Col-0 and Col-0 constitutively expressing cytosolic (apo)aequorin was as described in [19,20]. Growth conditions were as described by Matthus et al. in [19]. Growth medium was half-strength Murashige and Skoog (MS) with vitamins (Duchefa, Haarlem, The Netherlands) and 0.8% (*w*/*v*) agar (Bacto agar, BD Biosciences, San Jose, CA, USA), pH 5.6; “half MS”. This contained 0.625 mM phosphate (“full Pi”). A custom-made MS without Pi was used for “zero Pi” conditions (Duchefa, DU1072) and KCl substituted for missing potassium from KH_2_PO_4_ exclusion [19]. Plants were grown vertically or at a 45° incline.

### 4.2. Aequorin Luminometry

Excised whole roots or root tips of 10-day old seedlings were incubated overnight, in darkness at room temperature in 100 µL half MS containing 10 µM coelenterazine (NanoLight Technology, Pinetop, AZ, USA), pH 5.6 with MES (2-(*N*-morpholino)ethanesulfonic acid)/Tris (tris(hydroxymethyl)aminomethane) (Sigma-Aldrich Merck, Darmstadt, Germany). The half MS medium had the same nutrient status (i.e., full Pi or zero Pi) as that upon which the plants were grown. One root or root tip was placed per well (containing 100 µL of the appropriate full Pi or zero Pi half MS) in a white 96-well plate (Greiner Bio-One, Stroud, UK). Luminescence was recorded every second for 200 s in a bioluminescence plate reader (FLUOstar OPTIMA, BMG Labtech, Aylsebury, UK). After 35 s, 100 µL of full Pi or zero Pi half MS were added. Discharge solution (final concentration: 10% (*v*/*v*) ethanol, 1 M CaCl_2_) was injected after a further 120 s. Changes in [Ca^2+^]_cyt_ were estimated according to Knight et al. [6] and analysed according to Matthus et al. [19]; peak maxima were detected in a set time frame of 35–155 s. Total [Ca^2+^]_cyt_ mobilised was estimated as “Area Under the Curve” (AUC, [19]), after baseline subtraction. A summary schematic is shown in Figure 1a.

### 4.3. Root Skewing

After 8 days of vertical growth, the position of the root tip of each seedling was marked on the growth plate. Plants closest to the edge of the growth plate were not considered for analysis. Since plants on both full Pi as well as Pi-deficient conditions developed similarly up to day 7 (possibly due to seed reserves), marking the root tips on day 8 was considered as a point from where plant development diverged depending on the nutrient condition. After 11 days of growth, plates were scanned using an Epson scanner (Nagano, Japan) with 300 dpi resolution. The software, ImageJ, was used to quantify the root skewing angle, resulting from the root deviating away from vertical. The direction of root skewing was recorded, where growth to the right of vertical was considered as positive skewing and growth to the left as negative skewing, when viewed from the back of the plate. Root length was measured with the ImageJ plugin NeuronJ. During the test of growth at a 45° incline, plants were grown vertically for 6 days then root tip position was marked on the plate. Half the plates were grown vertically, and half the plates were placed at a 45° angle. The final scans were taken after a further 3 days.

### 4.4. Statistical Analyses

Statistical analyses were performed using the open-source software R (www.r-project.org, version 3.5.1) in an R studio environment. The ‘MESS’ (Miscellaneous Esoteric Statistical Scripts) package was used to calculate Area Under the Curve (AUC) in aequorin studies. A Welch two sample *t*-test was used to test for statistically significant differences, using a significance threshold of *p* < 0.05.
